# Just the facts: diagnosis and management of generalized pustular psoriasis

**DOI:** 10.1007/s43678-025-00883-9

**Published:** 2025-03-20

**Authors:** Jensen Yeung, Vimal H. Prajapati, Eric Mutter, Melinda Gooderham

**Affiliations:** 1 Department of Dermatology, Women’s College Hospital, Toronto, ON Canada; 2 Department of Dermatology, Sunnybrook Health Sciences Centre, Toronto, ON Canada; 3 Division of Dermatology, Department of Medicine, University of Toronto, Toronto, ON Canada; 4 Probity Medical Research, Waterloo, ON Canada; 5 Division of Dermatology, Department of Medicine, University of Calgary, Calgary, AB Canada; 6 Section of Community Pediatrics, Department of Pediatrics, University of Calgary, Calgary, AB Canada; 7 Section of Pediatric Rheumatology, Department of Pediatrics, University of Calgary, Calgary, AB Canada; 8 Dermatology Research Institute, Calgary, AB Canada; 9 Skin Health & Wellness Centre, Calgary, AB Canada; 10 Probity Medical Research, Calgary, AB Canada; 11 Department of Emergency Medicine, The Ottawa Hospital, Ottawa, ON Canada; 12 Department of Emergency Medicine, University of Ottawa, Ottawa, ON Canada; 13 SKiN Centre for Dermatology, Probity Medical Research and Queen’s University, Peterborough, ON Canada

**Keywords:** Generalized pustular psoriasis, Emergency department, Psoriasis pustuleuse généralisée, Service des urgences

## Clinical scenario

A 34-year-old female presents to the ED with the sudden appearance of a pruritic skin eruption comprised of pustules and erythema that started initially on her torso 2 weeks ago and now worsened to involve her extremities. She also reports skin pain, fever, fatigue, and tachycardia. Laboratory investigations show leukocytosis and C-reactive protein levels > 20 mg/L. A year ago, the patient developed a similar skin eruption which was successfully treated by her primary care provider with 2 weeks of prednisone after lack of response with topical corticosteroids.

## Key clinical questions

### What are the main pathogenic hallmarks of generalized pustular psoriasis?

Generalized pustular psoriasis is a rare and severe autoinflammatory skin disease characterized by an eruption of sterile, macroscopically visible, neutrophil-infiltrated pustules in a generalized fashion, although the trunk and proximal limbs are most commonly affected areas. Mucosal involvement may include persistent geographic or fissured tongue [1]. In contrast, localized pustular psoriasis primarily involves the hands and feet (palmoplantar pustular psoriasis), and no mucosal involvement. Although the full etiology of generalized pustular psoriasis remains unclear, triggers include infections, withdrawal of systemic corticosteroids, psychological stress, or pregnancy [1]. Affected areas in generalized pustular psoriasis are characterized by increased levels of pathogenic proinflammatory cytokines, primarily interleukin-36 (IL-36), which is a validated and clinically relevant therapeutic target [2, 3].

### What are the clinical features of generalized pustular psoriasis?

Generalized pustular psoriasis tends to develop abruptly, with potentially variable severity between flares. The initial cutaneous manifestations include erythema, edema, and pruritus, followed by formation of 2–3 mm sterile pustules within hours [3]. Pustules may also converge to form a ‘lake of pus’. Up to 65% of patients diagnosed with generalized pustular psoriasis will have a previous or concomitant plaque psoriasis diagnosis [3]. Therefore, pustules may also form within plaques. Pustules eventually dry out and disappear with residual crusting and desquamation, or may regress to erythrodermic psoriasis [3]. Generalized pustular psoriasis can be relapsing or persistent and is often accompanied by systemic inflammation. Non-cutaneous manifestations include headache, fever, fatigue, anorexia, nausea, tachycardia, uveitis, and arthritis [3].

### How is generalized pustular psoriasis diagnosed?

Urgent diagnosis and treatment of generalized pustular psoriasis is critical as it can lead to life-threatening complications, including bacterial superinfection, hemodynamic disorders, organ failure, or aseptic and hypovolemic shock [3, 4]. A prior plaque psoriasis diagnosis is helpful but not necessary to confirm generalized pustular psoriasis. A complete medication history is needed as abrupt withdrawal or rapid tapering of drugs such as systemic corticosteroids and cyclosporine can trigger generalized pustular psoriasis flares [1]. A complete physical examination of the skin and mucosae is required to assess disease severity. Laboratory investigations to confirm the diagnosis and evaluate for potential systemic inflammation include [1, 3]: complete blood count with differential (leukocytosis; lymphopenia), erythrocyte sedimentation rate (elevated), C-reactive protein (elevated), plasma globulins (IgG or IgA; elevated), albumin and calcium levels (hypoproteinemia and hypocalcemia), liver enzymes (elevated), urea and creatinine (elevated), and urinalysis (positive for albumin). Because generalized pustular psoriasis can be mistaken for an infection, pustule, blood, and urine bacterial cultures are recommended [1, 3, 5].

### What are the differential diagnoses of generalized pustular psoriasis?

Diagnosing generalized pustular psoriasis in the ED can be challenging, because it shares features with other pustular skin conditions, such as acute generalized exanthematous pustulosis and subcorneal pustular dermatosis [1, 3, 5]. Acute generalized exanthematous pustulosis is nonrecurrent and is characterized by pinhead-sized pustules arising within large areas of edematous skin. It is often triggered by drugs (especially systemic antibacterials, antifungals, and antimalarials) and accompanied by systemic signs/symptoms. This condition has a favorable prognosis after drug discontinuation. Subcorneal pustular dermatosis has a relapsing–remitting course and is characterized by flaccid pustules (several millimeters) that coalesce into annular or circinate patterns and typically occurs in elderly females. It is not usually accompanied by systemic signs/symptoms. Other generalized pustular psoriasis differential diagnoses include IgA pemphigus and pemphigus foliaceus [1, 5]. We recommend to immediately consult with a dermatologist for a definitive diagnosis [5].

### How is generalized pustular psoriasis managed in the ED?

It is important to first ensure that patients are hemodynamically stable by monitoring vital signs and administering fluid therapy if needed. The ED physician should seek an immediate dermatology consultation to confirm diagnosis and guide initial management [5]. The referral may be processed more urgently if it indicates suspected generalized pustular psoriasis. Inpatient admission may be warranted in case of severe generalized pustular psoriasis with significant systemic involvement. Specialties, including internal medicine, rheumatology, or ophthalmology, may also oversee care depending on disease manifestations. Generalized pustular psoriasis treatment follows already existing therapies for plaque psoriasis. The Joint 2020 AAD-NPF guidelines for plaque psoriasis recommend acitretin or cyclosporine as systemic non-biologic therapies for generalized pustular psoriasis. Second-line therapy for generalized pustular psoriasis includes plaque psoriasis-approved systemic biologic therapies (etanercept; infliximab; adalimumab; ustekinumab; secukinumab; ixekizumab; brodalumab), either as a monotherapy or in combination with systemic non-biologic therapies. Supportive care may include analgesics, antipyretics, and skin emollients. Caution should be exercised with rapid cessation of systemic corticosteroids or systemic biologics that target TNF-α, as this can result in generalized pustular psoriasis flares.

To date, intravenous spesolimab, an anti-IL-36R monoclonal antibody, is the only Health Canada-approved treatment of flares in adult generalized pustular psoriasis patients, while subcutaneous spesolimab is the only Health Canada-approved maintenance treatment for long-term management of adult generalized pustular psoriasis patients to minimize relapse. Use of off-label systemic non-biologic therapies can only temporarily reduce disease signs/symptoms, but they are associated with toxicity and disease recurrence, which limits their use as long-term maintenance options [6]. Biologics are, however, more costly than conventional systemics. Patient support programs are available for each biologic to guide patients through the healthcare system and explore available coverage options.

## Case resolution

The patient was initially diagnosed with an infection, admitted, and given empiric intravenous vancomycin and fluid replacement. A dermatologist was immediately consulted. Blood and pustule bacterial cultures were negative. Gram stain and KOH preparation also revealed no infection. The dermatologist performed a punch biopsy, which revealed Kogoj’s spongiform pustules and neutrophil infiltration in the stratum corneum supporting a generalized pustular psoriasis diagnosis. The patient was started on a single 900-mg intravenous dose of spesolimab infused over 90 min with almost complete clearance of pustules within 24 h. Four weeks later, the patient was started on subcutaneous spesolimab at a dose of 300 mg (two 150 mg injections) administered every 4 weeks to prevent future generalized pustular psoriasis flares.

## Key points

**Fig. 1:**
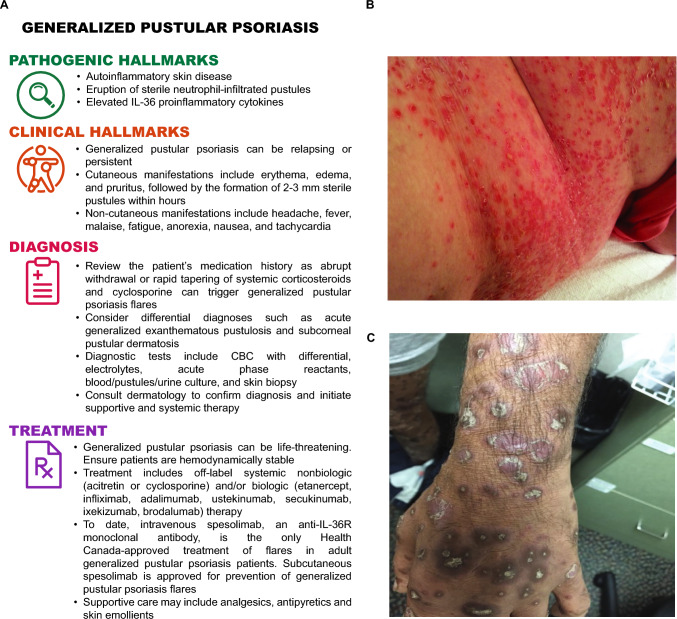
**A**. Pathogenesis, diagnosis, and treatment of generalized pustular psoriasis. **B**-**C**. Patient photographs showing pustules on the torso (**B**) and the left upper limb (**C**) at different stages of evolution and different skin tones

1. Generalized pustular psoriasis is a rare and severe autoinflammatory skin disease characterized by eruption of sterile, macroscopically visible pustules (Fig. 1).
2. Generalized pustular psoriasis can be relapsing or persistent and is often accompanied by systemic inflammation.
3. Generalized pustular psoriasis can be life-threatening and requires urgent care. Diagnostic tests include complete blood count with differential, electrolytes, acute phase reactants, blood/pustules/urine culture, and skin biopsy.
4. Dermatology should be immediately consulted to confirm the diagnosis and initiate the appropriate systemic non-biologic or biologic therapy.
